# Recent Advances in the Synthesis of Benzo[*d*]isothiazol-3(2*H*)-One and Benzo[*e*][1,3]Thiazin-4-One Derivatives

**DOI:** 10.3390/molecules30102099

**Published:** 2025-05-09

**Authors:** Yongli He, Dan Yuan, Ke Yang, Bindong Li

**Affiliations:** 1 School of Chemistry and Chemical Engineering, Nanjing University of Science and Technology, Nanjing 210094, China; hyl6080@njust.edu.cn; 2 Jiangsu Key Laboratory of Advanced Catalytic Materials & Technology, School of Petrochemical Engineering, Changzhou University, Changzhou 213164, China; s23020860015@smail.cczu.edu.cn

**Keywords:** benzo[*d*]isothiazol-3(2*H*)-ones, benzo[*e*][1,3]thiazin-4-ones, organic synthesis

## Abstract

Benzo[*d*]isothiazol-3(2*H*)-one and benzo[*e*][1,3]thiazin-4-one derivatives, as significant sulfur- and nitrogen-containing heterocyclic compounds, are prevalent in natural products, pharmaceuticals, and food items. In recent years, a variety of innovative synthetic methodologies for these compounds have been developed. In this review, we will comprehensively introduce the major advances in the synthesis of benzo[*d*]isothiazol-3(2*H*)-one and benzo[*e*][1,3]thiazin-4-one derivatives via both intramolecular and intermolecular pathways from 2012 to the present.

## 1. Introduction

Heterocyclic compounds are organic molecules that feature at least one ring structure composed of atoms from two or more different elements, typically carbon in combination with N, O, or S. These compounds play a fundamental role in both life processes and modern science, with applications extending across pharmaceuticals, biochemistry, agriculture, and materials science [1,2,3]. Benzo[*d*]isothiazol-3(2*H*)-one and benzo[*e*][1,3]thiazin-4-one derivatives, as important sulfur- and nitrogen-containing heterocyclic compounds with structural similarity, are extensively found in the fields of pharmaceutical chemistry, agriculture, and the food industry [4,5,6,7,8,9,10,11,12,13,14,15]. Consequently, the development of innovative and efficient methodologies for constructing these scaffolds has emerged as a focal point in these fields.

**Figure 1:**
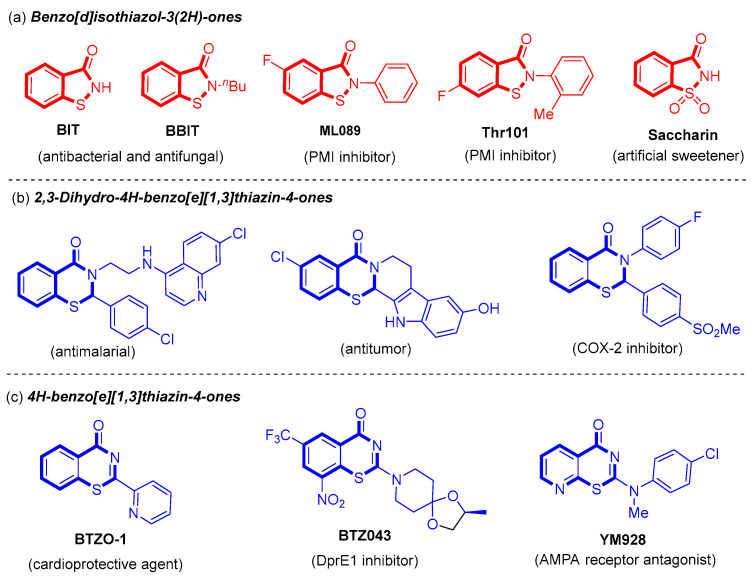
Some selected important benzo[*d*]isothiazol-3(2*H*)-one and benzo[*e*][1,3]thiazin-4-one derivatives.

Benzo[*d*]isothiazol-3(2*H*)-one derivatives are benzo-fused five-membered *N*,*S*-heterocycles (Figure 1a), whereas benzo[*e*][1,3]thiazin-4-one derivatives are benzo-fused six-membered *N*,*S*-heterocycles (Figure 1b,c). The key structural difference lies in the presence of an additional carbon atom between the N and S atoms in benzo[*e*][1,3]thiazin-4-one derivatives. Benzo[*d*]isothiazol-3(2*H*)-one derivatives represent a widely used scaffold in medicinal and agricultural compounds, exhibiting a broad spectrum of biological activities such as antibacterial, antifungal, antineoplastic, and hypoglycemic properties (Figure 1a) [4,5,6,7,8,9]. For example, benzo[*d*]isothiazol-3(2*H*)-one (BIT) and 2-butylbenzo[*d*]isothiazol-3(2*H*)-one (BBIT) can serve as crucial industrial biocides or cosmetic preservatives with antibacterial and antifungal properties [4,5,6]. 5-Fluoro-2-phenylbenzo[*d*]isothiazol-3(2*H*)-one (ML089) and 6-fluoro-2-(*o*-tolyl)benzo[*d*]isothiazol-3(2*H*)-one (Thr101) are potent inhibitors of phosphomannose isomerase (PMI) [7,8]. These compounds have demonstrated efficacy in anti-tumor applications and blood glucose regulation within the human body. Furthermore, saccharin, an oxidized derivative of benzisothiazol-3(2*H*)-one, is one of the most extensively used artificial sweeteners [9].

Benzo[*e*][1,3]thiazin-4-one derivatives, including 2,3-dihydro-4*H*-benzo[*e*][1,3]thiazin-4-ones (Figure 1b) and 4*H*-benzo[*e*][1,3]thiazin-4-ones (Figure 1c), have been identified in various natural products and pharmaceuticals [10,11,12,13,14,15]. Specifically, 2,3-dihydro-4*H*-benzo[*e*][1,3]thiazin-4-ones exhibit a diverse range of biological activities, including antimalarial, antitumor, and cyclooxygenase-2 (COX-2) inhibitory effects [10,11,12]. Moreover, 2-(pyridin-2-yl)-4*H*-benzo[*e*][1,3]thiazin-4-one (BTZO-1) has been recognized for its cardioprotective properties [13]. Additionally, 2-[(2*S*)-2-Methyl-1,4-dioxa-8-azaspiro [4.5]dec-8-yl]-8-nitro-6-(trifluoromethyl)-4*H*-1,3-benzothiazin-4-one (BTZ043) serves as an effective decaprenyl-phosphoribose-epimerase (DprE1) inhibitor that is crucial for tuberculosis treatment [14]. Furthermore, 2-[(4-chlorophenyl)(methyl)amino]-4*H*-pyrido [3,2-*e*][1,3]thiazin-4-one (YM928) is an orally active 2-amino-3-(3-hydroxy-5-methyl-4-isoxazolyl)propionic acid (AMPA) receptor antagonist for the treatment of various neurodegenerative diseases [15].

In recent years, various innovative methodologies for the synthesis of benzo[*d*]isothiazol-3(2*H*)-one and benzo[*e*][1,3]thiazin-4-one derivatives have been reported. Notably, in 2012, both the Xi group and the Punniyamurthy group independently pioneered a novel Cu(I)-catalyzed cascade reaction for the synthesis of benzo[*d*]isothiazol-3(2*H*)-ones [16,17]. Although considerable efforts have been made, only a few reviews and chapters have focused on the synthesis of these important skeletons [18,19,20,21,22,23,24]. In 2024, the Dehaen group summarized the recent advancements in the synthesis of benzo[*d*]isothiazol-3(2*H*)-ones [24]. In this review, we will introduce a comprehensive overview of the recent progress in the synthesis of benzo[*d*]isothiazol-3(2*H*)-one and benzo[*e*][1,3]thiazin-4-one derivatives via intramolecular and intermolecular pathways from 2012 to the present. Additionally, detailed discussions on the proposed mechanisms are included.

## 2. Synthesis of Benzo[d]isothiazol-3(2H)-Ones via Intramolecular and Intermolecular Pathways

### 2.1. Synthesis of Benzo[d]isothiazol-3(2H)-Ones via Intramolecular Pathways

**Scheme 1:**
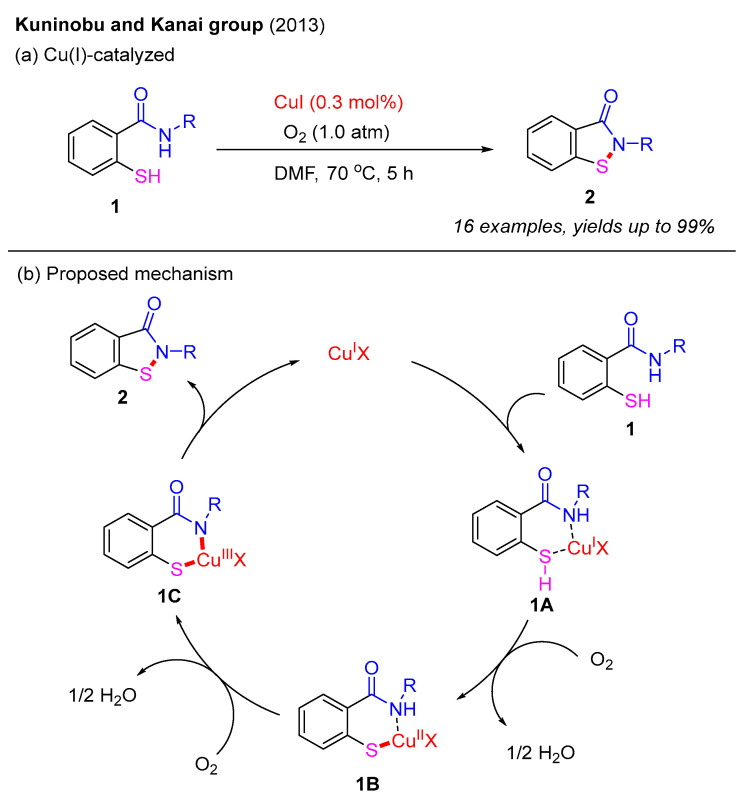
Cu(I)-catalyzed intramolecular N–S bond formation method to prepare benzo[*d*]isothiazol-3(2*H*)-ones under an O_2_ atmosphere [25].

The intramolecular construction of benzo[*d*]isothiazol-3(2*H*)-ones frequently employs 2-mercaptobenzamides as the starting materials. In 2013, the Kuninobu and Kanai group developed a Cu(I)-catalyzed intramolecular N–S bond formation method to synthesize benzo[*d*]isothiazol-3(2*H*)-ones under an O_2_ atmosphere (Scheme 1a). In this reaction, 2-mercaptobenzamides undergo intramolecular oxidative dehydrogenative cyclization, providing various benzo[*d*]isothiazol-3(2*H*)-ones in excellent yields. This strategy employs O_2_ as the sole oxidant, facilitating the coupling of N–H and S–H bonds, which led to the formation of a novel N–S bond. Furthermore, corresponding disulfides do not yield benzo[*d*]isothiazol-3(2*H*)-ones, suggesting that this process does not proceed via disulfide formation. Based on these observations, a proposed Cu(I)-catalyzed mechanism is illustrated in Scheme 1b. Initially, the coordination of 2-mercaptobenzamide **1** with Cu(I) catalyst generates intermediate **1A**, which is further oxidized by O_2_ to produce the Cu−S bond intermediate **1B**. Next, the second oxidation step provides the intermediate **1C**. Finally, reductive elimination of **1C** affords benzo[*d*]isothiazol-3(2*H*)-one **2** and regenerates the Cu(I) catalyst [25].

**Scheme 2:**
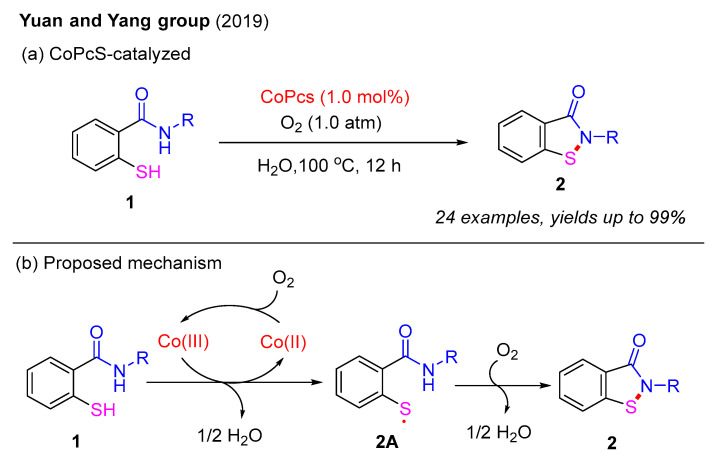
CoPcS-catalyzed intramolecular oxidative dehydrogenative cyclization for the synthesis of benzo[*d*]isothiazol-3(2*H*)-ones under an O_2_ atmosphere [26].

In 2019, the Yuan and Yang group employed a heterogeneous catalyst, *tetra*-substituted sulfonated cobalt phthalocyanine (CoPcS), to synthesize benzo[*d*]isothiazol-3(2*H*)-ones from 2-mercaptobenzamides under an O_2_ atmosphere in aqueous media (Scheme 2a). A wide range of benzo[*d*]isothiazol-3(2*H*)-ones were isolated in good to excellent yields. Notably, the use of H_2_O as the reaction solvent not only facilitates product purification but also enables the recycling of the mother liquor. A proposed mechanism is outlined in Scheme 2b. The initial oxidation of Co(II) by O_2_ forms the Co(III) catalyst, which oxidizes 2-mercaptobenzamide **1** to produce the thiyl radical intermediate **2A** while regenerating the Co(II) complex. Subsequently, under an O_2_ atmosphere, intramolecular nucleophilic attack by the N–H bond on the sulfur atom in intermediate **2A** leads to the formation of the final product **2** [26].

**Scheme 3:**
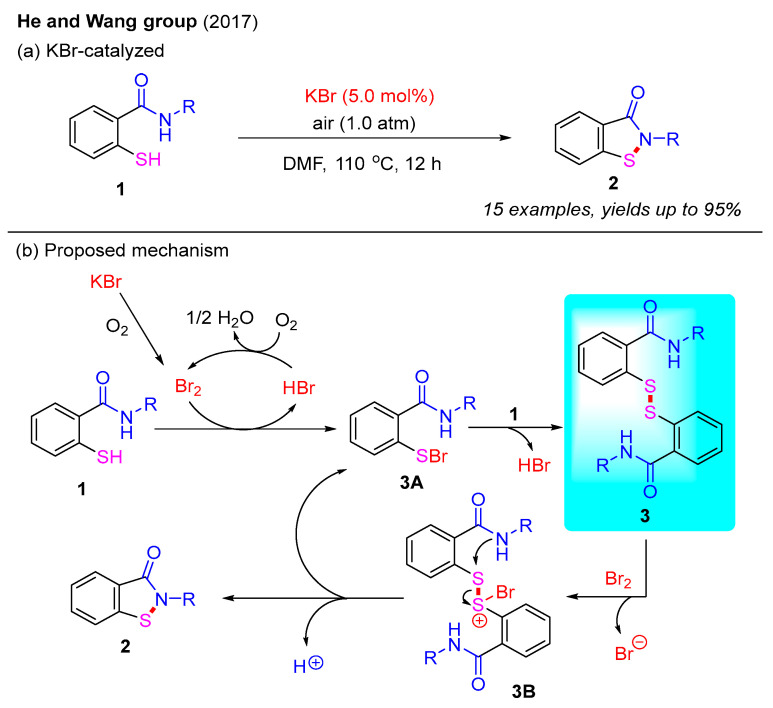
KBr-catalyzed intramolecular oxidative dehydrogenative cyclization for the synthesis of benzo[*d*]isothiazol-3(2*H*)-ones under an O_2_ atmosphere [27].

In addition to transition-metal catalysis strategies, metal-free approaches have garnered significant attention due to their green and sustainable properties. In 2017, the He and Wang group reported a KBr-catalyzed intramolecular oxidative dehydrogenative cyclization for constructing benzo[*d*]isothiazol-3(2*H*)-ones under an O_2_ atmosphere (Scheme 3a) [27]. This reaction successfully transformed 2-mercaptobenzamides into the desired benzo[*d*]isothiazol-3(2*H*)-ones in excellent yields. The structures of the products were confirmed using X-ray crystallography. Furthermore, mechanistic studies indicate that disulfides may be involved in this process. Based on these findings, a reaction mechanism is proposed in Scheme 3b. First, KBr is oxidized to Br_2_ under an O_2_ atmosphere. Next, 2-mercaptobenzamide **1** reacts with Br_2_ to form intermediate **3A** and HBr. Notably, HBr can be re-oxidized by O_2_ to regenerate Br_2_, thereby maintaining the catalytic cycle. Meanwhile, the crucial intermediate **3A** can react with 2-mercaptobenzamide to form disulfide intermediate **3** via the elimination of HBr. Next, disulfide intermediate **3** is activated by Br_2_ to generate intermediate **3B**, which can further convert into the final product **2** and regenerate intermediate **3A** [28].

**Scheme 4:**
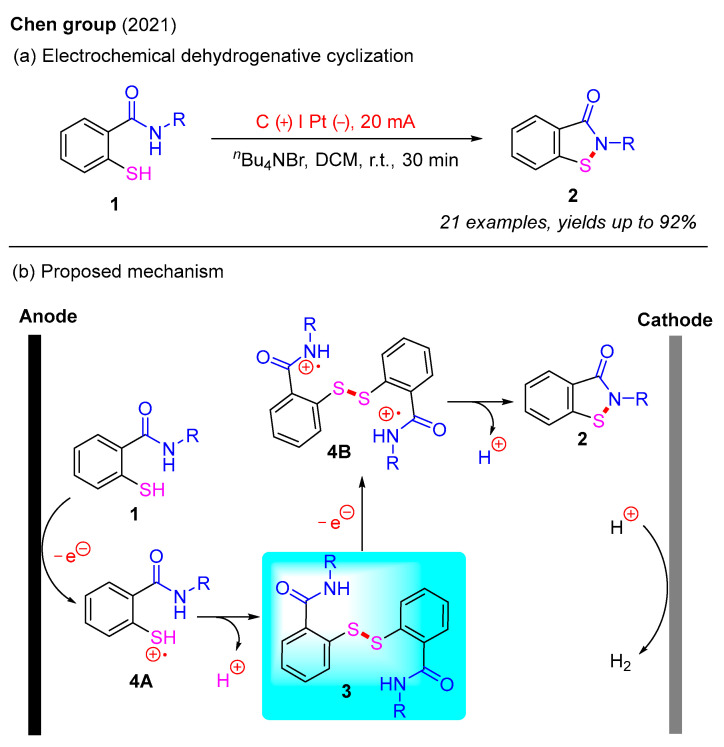
Electrochemical intramolecular dehydrogenative cyclization for the synthesis of benzo[*d*]isothiazol-3(2*H*)-ones [31].

Recently, electrochemistry has emerged as a powerful and versatile tool in the field of organic synthesis. Electricity is gaining recognition as a green and sustainable redox agent because it serves as an ideal alternative to conventional chemical oxidants or reductants [29,30]. In 2021, the Chen group reported an electrochemical dehydrogenative cyclization protocol for the synthesis of benzo[*d*]isothiazol-3(2*H*)-ones via intramolecular N–S bond formation (Scheme 4a) [31]. In this study, various benzo[*d*]isothiazol-3(2*H*)-ones were isolated in moderate to good yields using 2-mercaptobenzamides as starting materials and tetrabutylammonium bromide [(*n*-Bu)_4_NBr] as an additive. Furthermore, this process was carried out through constant-current electrolysis in an undivided cell, with H_2_ as the nonhazardous byproduct. A proposed electrochemical mechanism is illustrated in Scheme 4b. Initially, the oxidation of 2-mercaptobenzamide **1** at the anode generates intermediate **4A**, which subsequently undergoes intermolecular coupling and deprotonation to yield the disulfide intermediate **3**. Next, the anodic oxidation of intermediate **3** provides intermediate **4B**, which is converted into the final product **2** via intramolecular cyclization and deprotonation [32].

In recent years, Selectfluor [1-chloromethyl-4-fluoro-1,4-diazoniabicyclo-[2.2.2]octane bis(tetrafluoroborate)] has emerged not only as a crucial electrophilic fluorine reagent but also as a versatile “fluorine-free” reagent in various organic reactions [33,34,35,36,37].

**Scheme 5:**
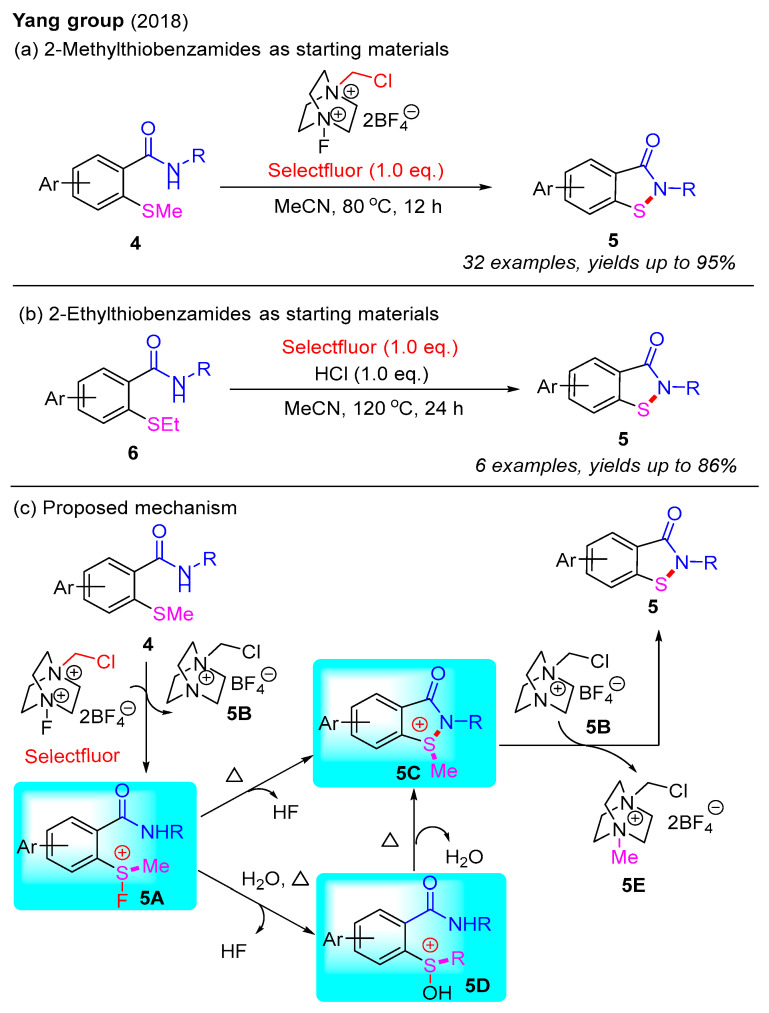
Selectfluor-mediated intramolecular N–S bond formation for the synthesis of benzo[*d*]isothiazol-3(2*H*)-ones [38].

In 2018, the Yang group pioneered the use of Selectfluor for the synthesis of benzo[*d*]isothiazol-3(2*H*)-ones via a cascade N–S bond formation and the C(sp^3^)–S bond cleavage of 2-methylthiobenzamides (Scheme 5a) [38]. They subsequently found that the addition of HCl facilitated the conversion of 2-ethylthiobenzamides into benzo[*d*]isothiazol-3(2*H*)-ones (Scheme 5b) [39]. In these reactions, Selectfluor serves as a source of the fluorine cation (F^+^), which activates the methylthio group to form a transient fluorosulfonium salt. A plausible mechanism for this is presented in Scheme 5c. Initially, 2-methylthiobenzamide **4** reacts with Selectfluor to generate a transient fluorosulfonium salt **5A** and an intermediate salt **5B.** Subsequently, the fluorosulfonium salt **5A** can either directly cyclize to form the cyclic sulfonium salt **5C**, or it can be converted into salt **5C** via the intermediate formation of salt **5D**. Finally, benzo[*d*]isothiazol-3(2*H*)-one **5** is synthesized through nucleophilic substitution of salt **5C** by salt **5B**.

### 2.2. Synthesis of Benzo[d]isothiazol-3(2H)-Ones via Intermolecular Pathways

In addition to intramolecular pathways, 2-halobenzamides can undergo intermolecular reactions with sulfur powder (S_8_), potassium thiocyanate (KSCN), and carbon disulfide (CS_2_) to construct benzo[*d*]isothiazol-3(2*H*)-ones in the presence of transition-metal catalysts.

**Scheme 6:**
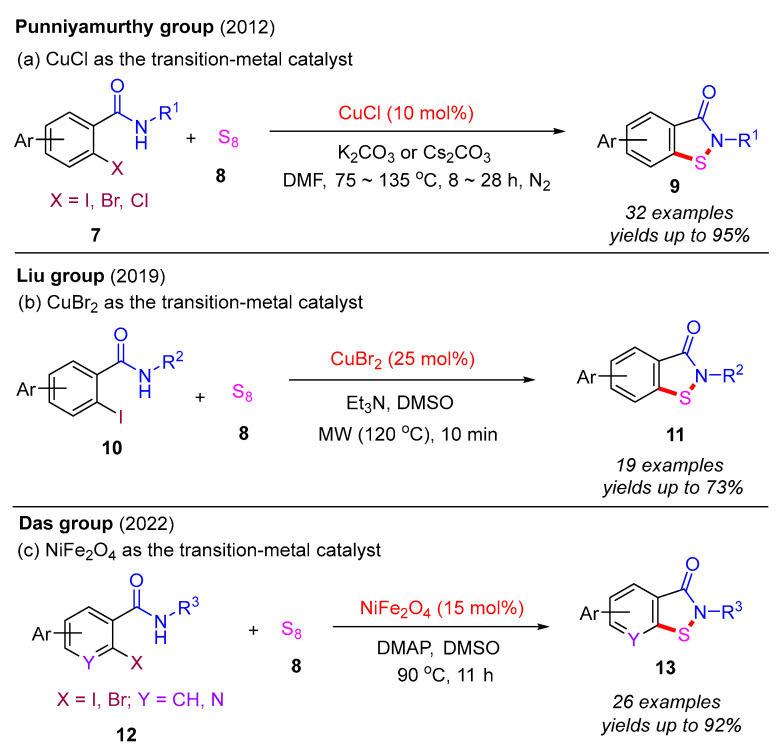
Transition-metal-catalyzed cascade reaction involving C–S bond formation followed by N–S bond cyclization for the synthesis of benzo[*d*]isothiazol-3(2*H*)-ones from 2-halobenzamides and S_8_ [16,40,41].

**Scheme 7:**
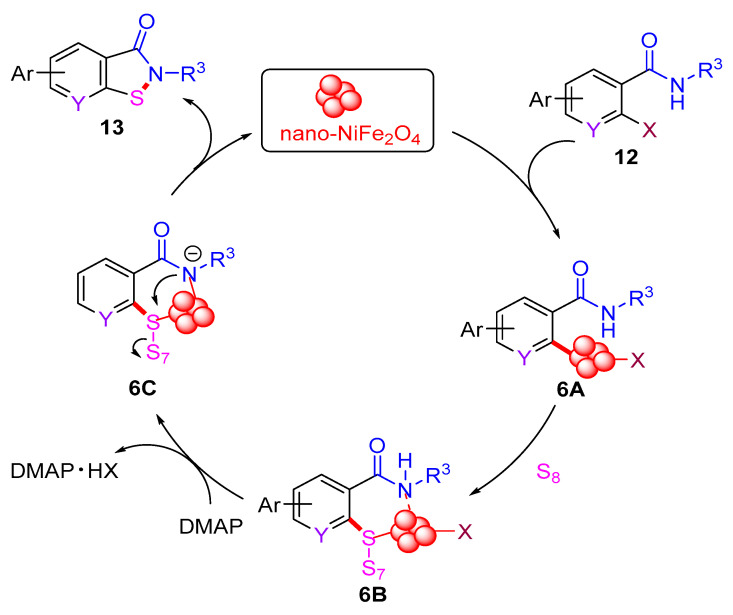
The plausible mechanism of nano-NiFe_2_O_4_-catalyzed cascade reaction involving C–S bond formation followed by N–S bond cyclization.

In 2012, the Punniyamurthy group developed a CuCl-catalyzed cascade reaction involving C–S bond formation followed by N–S bond cyclization (Scheme 6a) [16]. This protocol enabled the synthesis of benzo[*d*]isothiazol-3(2*H*)-ones in moderate to good yields via the coupling of various 2-halobenzamides with S_8_. The reactivity order of 2-halobenzamides follows the sequence 2-iodobenzamide > 2-bromobenzamide > 2-chlorobenzamide. Subsequently, in 2019, the Liu group employed the CuBr_2_-catalyzed approach to synthesize benzo[*d*]isothiazol-3(2*H*)-ones from 2-iodobenzamides and S_8_ under microwave irradiation (MW) (Scheme 6b) [40]. In 2022, the Das group used a recyclable nano-nickel ferrite catalyst (nano-NiFe_2_O_4_) to perform this cascade reaction, employing 2-halobenzamides with S_8_ as starting materials (Scheme 6c) [41]. A plausible mechanism for this process is proposed (Scheme 7). Initially, 2-halobenzamide **12** reacts with nano-NiFe_2_O_4_ to form intermediate **6A**, which then reacts with S_8_ to yield intermediate **6B**. In the presence of DMAP, intermediate **6B** undergoes transformation into **6C** via the elimination of DMAP·HX. Finally, the cyclization of the N–S bond in intermediate **6C** yields the desired product **13** and regenerates the catalyst nano-NiFe_2_O_4_ [42].

**Scheme 8:**
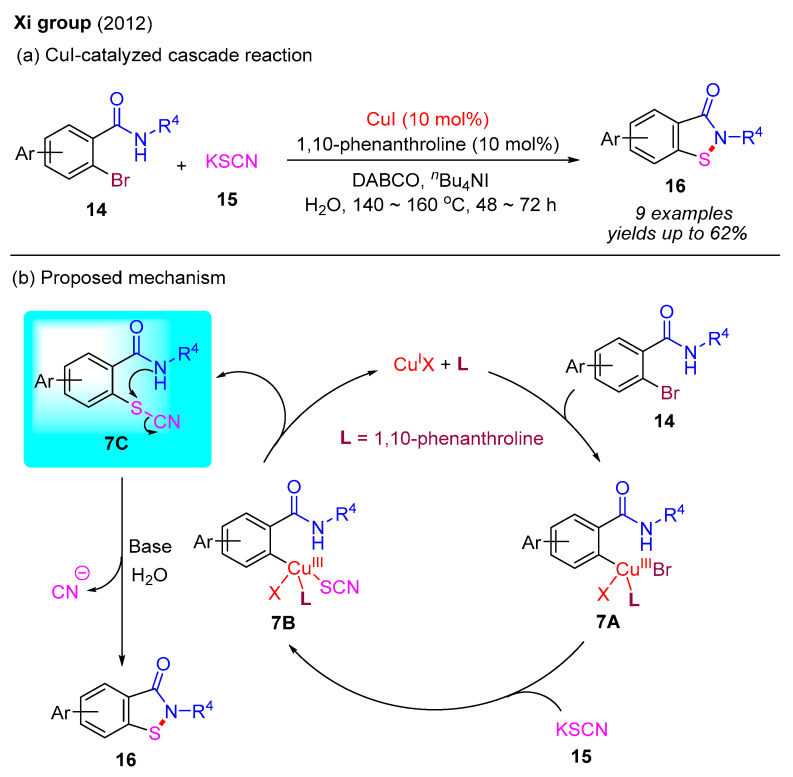
CuI-catalyzed cascade reaction for the synthesis of benzo[*d*]isothiazol-3(2*H*)-ones using 2-bromobenzamides and KSCN [17].

In 2012, the Xi group employed KSCN as the sulfur source to react with 2-bromobenzamides, constructing benzo[*d*]isothiazol-3(2*H*)-ones via a similar Cu(I)-catalyzed cascade reaction with C–S bond and N–S bond formation in an aqueous medium (Scheme 8a) [17]. In this process, 1,10-phenanthroline served as the ligand, DABCO functioned as the base, and Bu_4_NI was utilized as an additive. Notably, this reaction required higher temperatures and extended reaction times, leading to only moderate yields of the desired products. A plausible reaction mechanism is provided as shown in Scheme 8b. The initial oxidative addition between 2-bromobenzamide **14** with the Cu(I) catalyst generates intermediate **7A**, which subsequently undergoes ligand exchange with KSCN to form intermediate **7B**. The ensuing reductive elimination of **7B** produces thiocyanate intermediate **7C** and regenerates the Cu(I) catalyst. In the presence of a base and H_2_O, intermediate **7C** is converted into the final product **16** via an intramolecular nucleophilic substitution reaction. Concurrently, the in situ generated CN anion can be hydrolyzed by the base during this process.

**Scheme 9:**
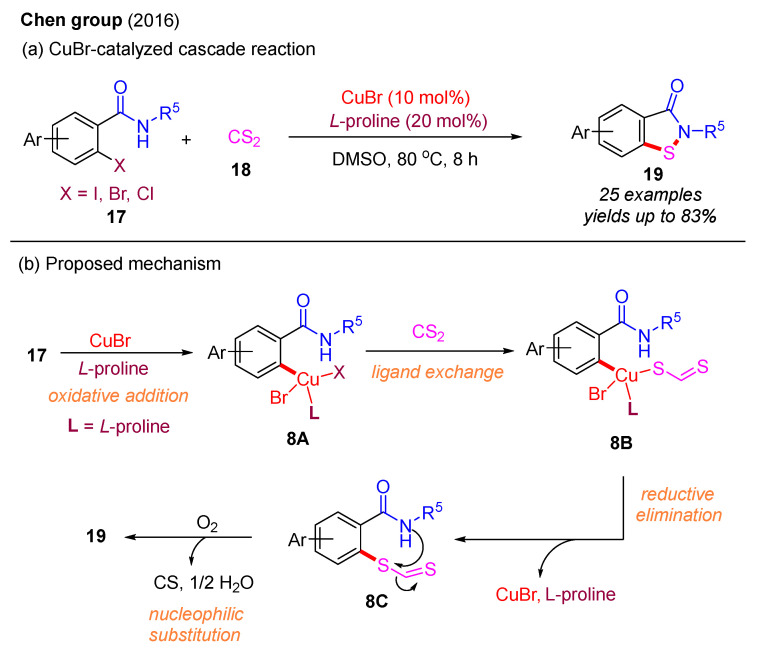
CuBr-catalyzed cascade reaction with C−S bond and N−S bond formation using 2-halobenzamides and CS_2_ [43].

Subsequently, in 2016, the Chen group reported that CS_2_ could serve as a sulfur source to react with 2-halobenzamides for the preparation of benzo[*d*]isothiazol-3(2*H*)-ones under a Cu(I)-catalyzed system (Scheme 9a). Over 25 examples were synthesized with moderate to good yields. Notably, *L*-proline was identified as the optimal ligand, exhibiting superior catalytic performance compared to 1,10-phenanthroline. The reaction mechanism involves an initial oxidative addition, yielding intermediate **8A**, which is followed by ligand exchange to form intermediate **8B**. Subsequent reductive elimination of **8B** affords the intermediate **8C**, which then undergoes nucleophilic substitution to provide the desired product **19** (Scheme 9b) [43].

**Scheme 10:**
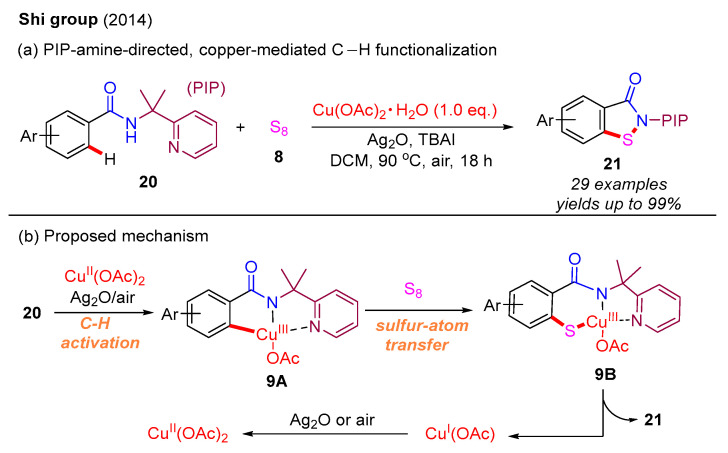
PIP-amine-directed, copper-mediated C–H functionalization strategy for the synthesis of benzo[*d*]isothiazol-3(2*H*)-ones [50].

Recently, C–H functionalization has emerged as an exceptionally efficient method for constructing C–C and C–heteroatom bonds [44,45,46,47,48,49]. In 2014, the Shi group first developed a PIP-amine [(pyridin-2-yl)isopropylamine]-directed, copper-mediated C–H functionalization strategy to construct benzo[*d*]isothiazol-3(2*H*)-ones. Within this approach, various benzamides react with S_8_ to produce the desired products, with good yields, via C–S bond and N–S bond formation (Scheme 10a). Based on mechanistic studies and previous work, the authors propose that the benzamide substrate **20** undergoes initial C–H functionalization to form intermediate **9A** in the presence of Cu(II) species, Ag_2_O, and air. This intermediate is subsequently transformed into intermediate **9B** through a sulfur-atom transfer. Following this, the N–S reductive elimination of **9B** yields the final product **21** along with Cu(I) species. Importantly, the Cu(I) species is re-oxidized by Ag_2_O or air to regenerate Cu(II) species (Scheme 10b) [50].

## 3. Synthesis of 2,3-Dihydro-4H-Benzo[e][1,3]Thiazin-4-Ones via Intramolecular and Intermolecular Pathways

### 3.1. Synthesis of 2,3-Dihydro-4H-Benzo[e][1,3]Thiazin-4-Ones via Intramolecular Pathways

**Scheme 11:**
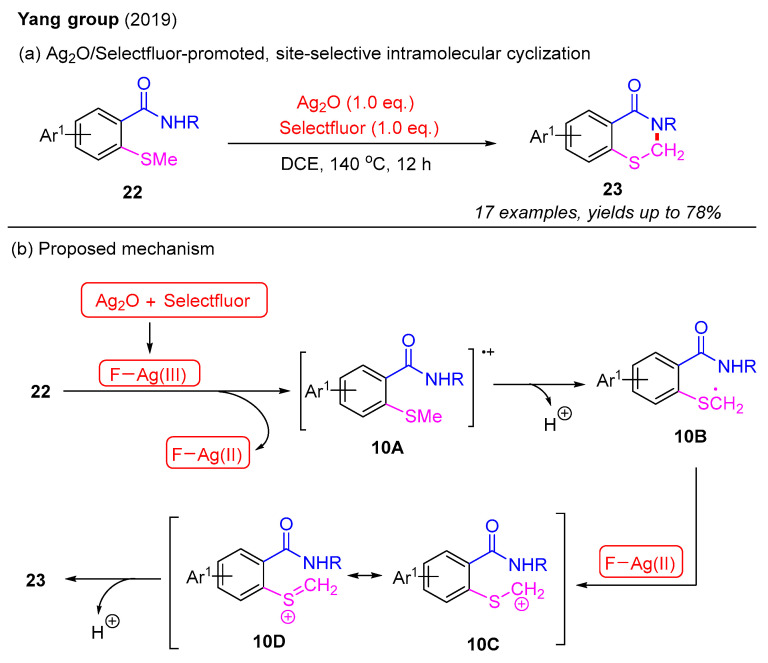
Ag_2_O/Selectfluor-promoted, site-selective intramolecular cyclization of 2-methylthiobenzamides for the synthesis of 2,3-dihydro-4*H*-benzo[*e*][1,3]thiazin-4-ones [51].

It is imperative to highlight that the literature on the intramolecular synthesis of 2,3-dihydro-4*H*-benzo[*e*][1,3]thiazin-4-ones remains limited. In 2019, the Yang group introduced an Ag_2_O/Selectfluor-promoted, site-selective intramolecular cyclization of 2-methylthiobenzamides to synthesize 2,3-dihydro-4*H*-benzo[*e*][1,3]thiazin-4-ones (Scheme 11a). In this process, the α-C(sp^3^)–H bond functionalization of the methyl group yielded various 2,3-dihydro-4*H*-benzo[*e*][1,3]thiazin-4-ones in good yields. However, attempts to substitute the methyl group on the sulfur atom with an ethyl group failed to produce the desired product. A plausible mechanism for this is proposed in Scheme 11b. The initial oxidation between Ag_2_O and Selectfluor forms the F–Ag(III) species, which further reacts with 2-methylthiobenzamide **22** to produce intermediate **10A** and Ag(II)–F species. The subsequent deprotonation of **10A** leads to the formation of intermediate **10B**. The second oxidation of **10B** by the Ag(II)–F species provides intermediate **10C** and its resonant intermediate **10D**. Finally, intramolecular cyclization of either **10C** or **10D** results in the formation of the desired product **23** [51].

**Scheme 12:**
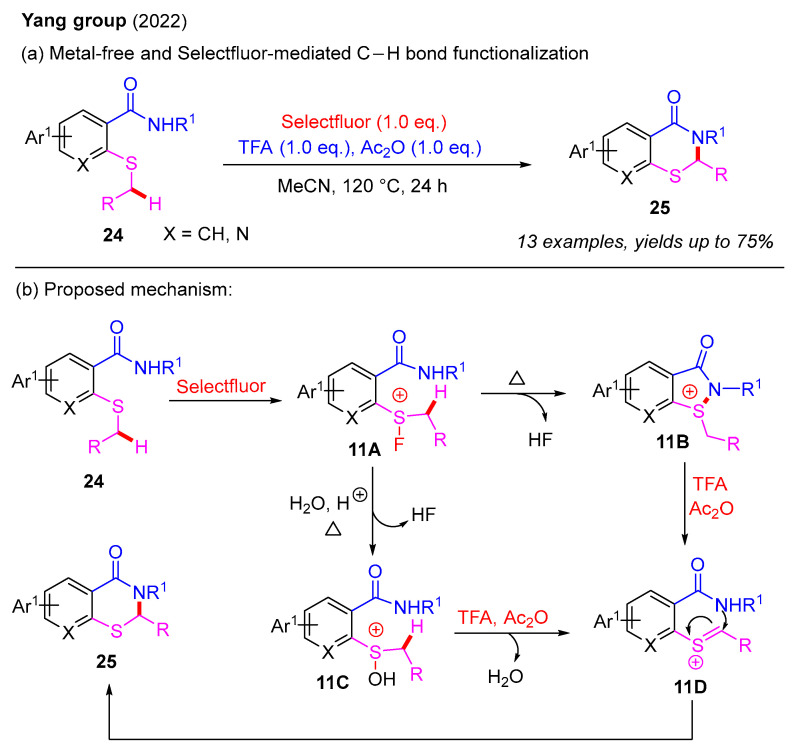
Metal-free and Selectfluor-mediated α-C(sp^3^)–H bond functionalization of 2-alkylthiobenzamides for the synthesis of 2-substituted 2,3-dihydro-4*H*-benzo[*e*][1,3]thiazin-4-ones [39].

In 2022, the same group further advanced this field by developing a metal-free and Selectfluor-mediated α-C(sp^3^)–H bond functionalization of 2-alkylthiobenzamides to construct 2-substituted 2,3-dihydro-4*H*-benzo[*e*][1,3]thiazin-4-ones (Scheme 12a). In this process, trifluoroacetic acid (TFA) and acetic anhydride (Ac_2_O) played critical roles as additives. Moreover, Selectfluor acted as a F^+^ initiator to activate the thioether group to facilitate this reaction. A proposed reaction pathway is also provided in Scheme 12b. Firstly, 2-alkylthiobenzamide **24** reacts with Selectfluor to form fluorosulfonium salt **11A**. Next, fluorosulfonium salt **11A** can be converted into either cyclic sulfonium salt **11B** or hydroxy sulfonium intermediate **11C**. In the presence of TFA and Ac_2_O, sulfonium salt **11D** is generated from intermediates **11B** or **11C**. Finally, the intramolecular cyclization of **11D** affords the desired product **25** [39].

### 3.2. Synthesis of 2,3-Dihydrobenzothiazin-4-Ones via Intermolecular Pathways

**Scheme 13:**
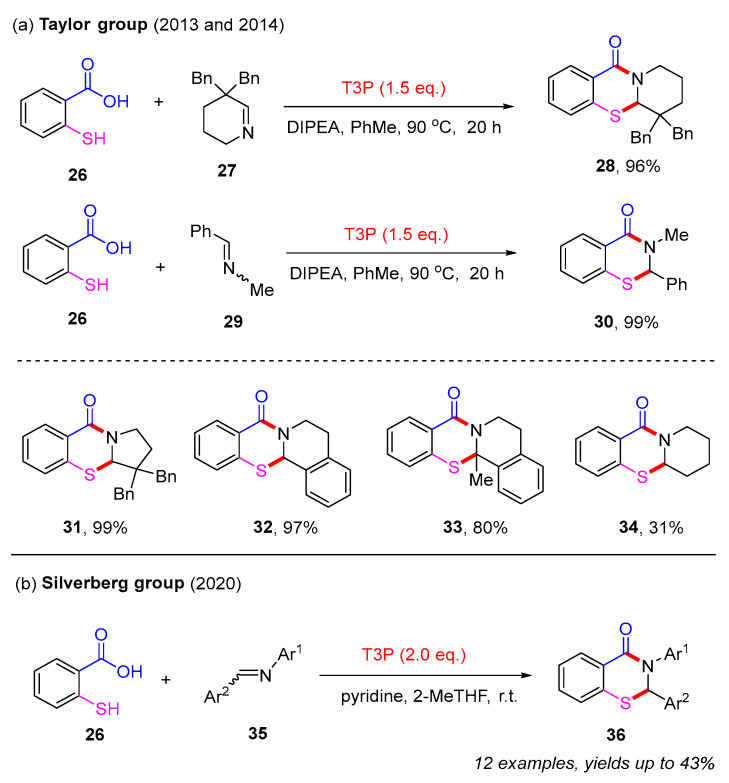
The synthesis of 2,3-dihydro-4*H*-benzo[*e*][1,3]thiazin-4-ones from 2-mercaptobenzoic acids and imines [52,53,54].

In contrast to the limited intramolecular pathways, the synthesis of 2,3-dihydro-4*H*-benzo[*e*][1,3]thiazin-4-ones primarily depends on intermolecular strategies. In 2013, the Taylor group reported a simple and direct imine acylation approach for the synthesis of 2,3-dihydro-4*H*-benzo[*e*][1,3]thiazin-4-ones. By utilizing 2-mercaptobenzoic acid **26** and cyclic imine **27** as starting materials, the desired product **28** was isolated in a 96% yield in the presence of T3P (propylphosphonic acid anhydride) and DIPEA (diisopropylethylamine). Additionally, acylic imine **29** was also found to produce product **30** with excellent yield (Scheme 13a). Mechanistic studies suggested that this process likely proceeds via the initial formation of an *N*-acyliminium ion, which is subsequently captured by an intramolecular S–H bond [52].

In subsequent studies, the Taylor group extended this approach to various cyclic imines in combination with 2-mercaptobenzoic acid. Different 2,3-dihydro-4*H*-benzo[*e*][1,3]thiazin-4-ones **31**–**33** were isolated in good to excellent yields. However, product **34** was only obtained in 31% yield due to this kind of imine existing primarily in its trimeric form, dodecahydro-4a,8a,12a-triazatriphenylene (Scheme 13a) [53]. In 2020, the Silverberg group used diaryl imines to react with 2-mercaptobenzoic acids in the presence of T3P. However, this strategy only afforded a limited range of 2,3-dihydro-4*H*-benzo[*e*][1,3]thiazin-4-ones **36** with low yields (Scheme 13b) [54].

**Scheme 14:**
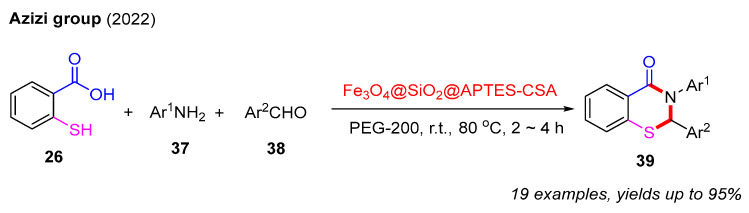
The synthesis of 2,3-dihydro-4*H*-benzo[*e*][1,3]thiazin-4-ones via a three-component reaction involving 2-mercaptobenzoic acids, aldehydes, and amines [55].

To our delight, in 2022, the Azizi group successfully developed a recyclable heterogeneous catalyst, Fe_3_O_4_@SiO_2_@APTES-CSA. This catalyst was employed to achieve the efficient synthesis of 2,3-dihydro-4*H*-benzo[*e*][1,3]thiazin-4-ones via a three-component reaction involving 2-mercaptobenzoic acids, aldehydes, and amines in the green solvent PEG-200. Notably, this catalyst can be reused at least five times without any observable decrease in its catalytic performance (Scheme 14) [55].

**Scheme 15:**
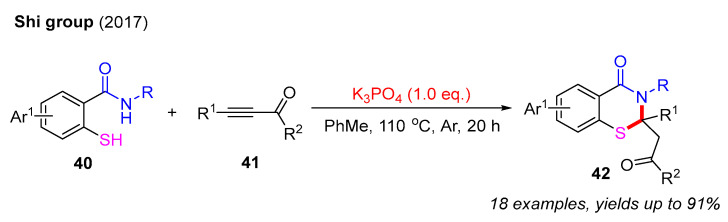
The synthesis of 2,3-dihydro-4*H*-benzo[*e*][1,3]thiazin-4-ones using 2-mercaptobenzamides with propiolate derivatives [56].

In 2017, the Shi group successfully synthesized a series of 2,3-dihydro-4*H*-benzo[*e*][1,3]thiazin-4-ones using 2-mercaptobenzamides and propiolate derivatives for preparation in the presence of K_3_PO_4_. This reaction yielded a diverse range of products **42** with satisfactory yields. Mechanistic studies revealed that the initial 1,4-addition between 2-mercaptobenzamides and propiolate derivatives provides alkene intermediates that undergo intramolecular cyclization under basic conditions to form the final products (Scheme 15) [56].

**Scheme 16:**
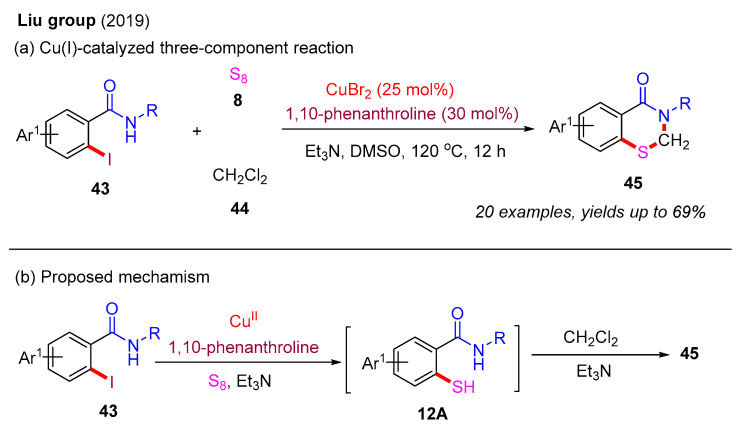
Cu(I)-catalyzed three-component reaction of 2-iodobenzamides, S_8_, and DCM for the construction of 2,3-dihydro-4*H*-benzo[*e*][1,3]thiazin-4-ones [40].

In addition to 2-mercaptobenzamides and 2-mercaptobenzoic acids, 2-iodobenzamides can also serve as effective precursors for the construction of 2,3-dihydro-4*H*-benzo[*e*][1,3]thiazin-4-ones. In 2019, the Liu group reported a Cu(I)-catalyzed three-component reaction involving 2-iodobenzamides, S_8_, and dichloromethane (DCM) (Scheme 16a). This protocol enabled the synthesis of various 2,3-dihydrobenzothiazin-4-ones in moderate to good yields through a cascade thiolation and annulation process. Notably, 1,10-phenanthroline was identified as the optimal ligand, significantly enhancing the reaction yield. A more reasonable proposed mechanism is presented in Scheme 16b. In the presence of a Cu(II) catalyst, 1,10-phenanthroline, Et_3_N, and S_8_, 2-iodobenzamide **43** is first converted into 2-mercaptobenzamide **12A** via a typical Ullmann-type thiolation reaction. Next, 2-mercaptobenzamide **12A** can directly react with DCM in the presence of Et_3_N to produce the desired product **45** [40].

**Scheme 17:**
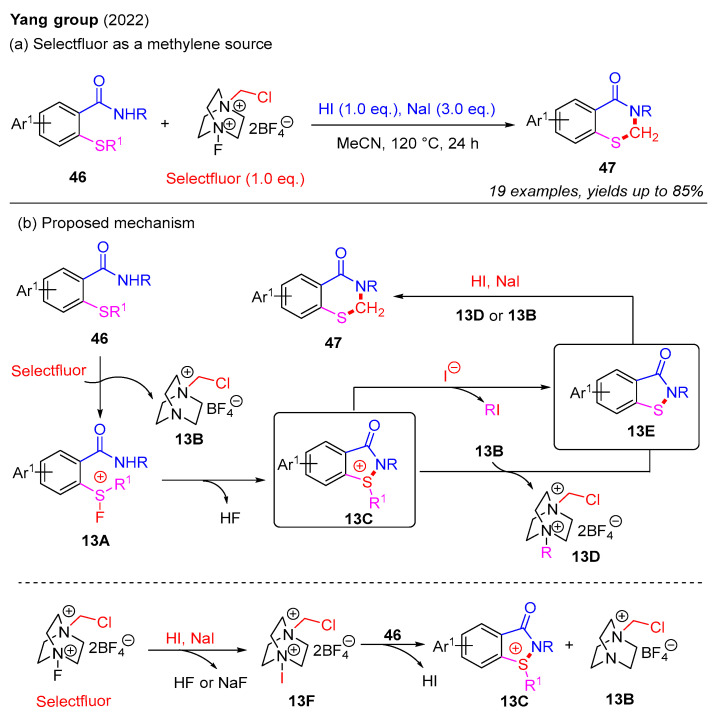
The use of Selectfluor as a methylene source for the construction of 2,3-dihydro-4*H*-benzo[*e*][1,3]thiazin-4-ones from 2-alkylthiobenzamides in the NaI-HI system [39].

In 2022, the Yang group utilized Selectfluor as a methylene source to synthesize 2,3-dihydrobenzothiazin-4-ones from 2-alkylthiobenzamides in the NaI-HI system (Scheme 17a) [39]. This process featured the selective cleavage of C(sp^3^)–S bonds in 2-alkylthiobenzamides, resulting in the formation of various 2,3-dihydro-4*H*-benzo[*e*][1,3]thiazin-4-ones with satisfactory yields. Furthermore, 2-alkylthiobenzamides bearing diverse sulfur substituents, including *n*-propyl, *n*-butyl, benzyl, isopropyl, and methyl groups, were well-tolerated in this reaction, affording the desired products in good yields. Mechanistic studies indicated that Selectfluor functions as a bifunctional reagent, serving both F^+^ initiator and methylene sources in the NaI-HI system. A plausible mechanism for this is presented in Scheme 17b. Initially, the reaction between 2-alkylthiobenzamide **46** and Selectfluor forms a transient fluorosulfonium salt **13A** and **a** chloromethyl salt **13B**. Subsequently, intramolecular cyclization of **13A** produces sulfonium salt **13C**. Next, nucleophilic displacement of **13C** by **13B** or an iodide ion affords intermediate **13E** in the presence of NaI and HI. Finally, ring expansion between intermediate **13E** and **13D** or **13B** provides the desired product **47**. Moreover, it is also proposed that Selectfluor in the NaI-HI system may generate intermediate **13F**, which reacts with **46** to form intermediate **13C** and chloromethyl salt **13B**.

**Scheme 18:**
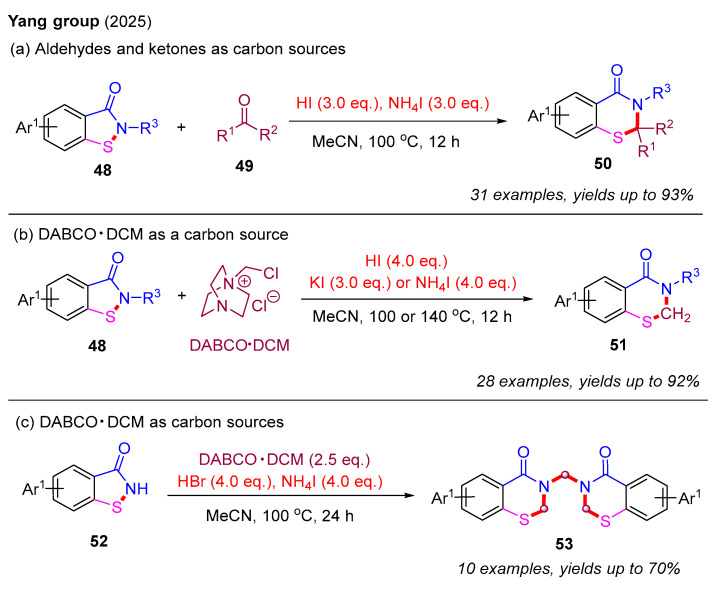
The metal-free ring-expansion strategy for the construction of 2,3-dihydro-4*H*-benzo[*e*][1,3]thiazin-4-ones through a single-carbon insertion approach [57].

Recently, the same group also developed a novel metal-free ring-expansion strategy to construct 2,3-dihydro-4*H*-benzo[*e*][1,3]thiazin-4-ones from benzoisothiazol-3-ones through a single-carbon insertion approach. In this strategy, both 2-substituted and 2,2-disubstituted 2,3-dihydro-4*H*-benzo[*e*][1,3]thiazin-4-ones **50** were isolated in good to excellent yields. Specifically, in the presence of HI and NH_4_I, aldehydes and ketones served as single-carbon sources for the selective formation of 2-substituted and 2,2-disubstituted 2,3-dihydrobenzothiazin-4-ones, respectively (Scheme 18a). Notably, DABCO·DCM [1-(chloromethyl)-1,4-diazabicyclo [2.2.2]octan-1-ium chloride] was utilized for the first time as a single-carbon source for constructing 2,3-dihydro-4*H*-benzo[*e*][1,3]thiazin-4-ones **51** in the presence of HI and KI or NH_4_I (Scheme 18b). Furthermore, bis-2,3-dihydro-4*H*-benzo[*e*][1,3]thiazin-4-ones **53** were synthesized in moderate to good yields using DABCO·DCM in the presence of HBr and NH_4_I (Scheme 18c) [57].

**Scheme 19:**
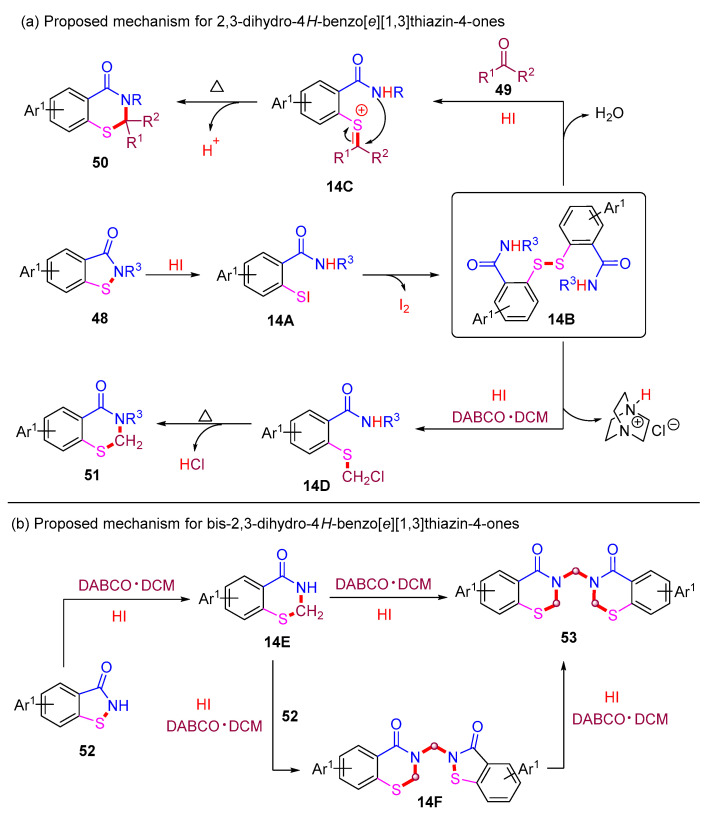
Proposed mechanisms for 2,3-dihydro-4*H*-benzo[*e*][1,3]thiazin-4-ones via metal-free ring-expansion strategies.

The proposed reaction mechanisms for 2,3-dihydro-4*H*-benzo[*e*][1,3]thiazin-4-ones are depicted in Scheme 19a. In the presence of HI, the initial ring-opening of benzoisothiazol-3-one **48** proceeds to form intermediate **14A**. Subsequently, intermediate **14A** undergoes rapid transformation into the crucial disulfide intermediate **14B**. Next, intermediate **14B** reacts with an aldehyde or ketone to form intermediate **14C**, which subsequently undergoes intramolecular cyclization to yield the desired product **50**. On the other hand, the reaction between **14B** and DABCO·DCM affords intermediate **14D,** which is further transformed into the desired product **51** through HCl elimination.

The proposed mechanism for the construction of bis-2,3-dihydro-4*H*-benzo[*e*][1,3]thiazin-4-ones is provided in Scheme 19b. The HI-mediated ring-expansion of benzoisothiazol-3-one **52** with DABCO·DCM generates intermediate **14E**, which further reacts with DABCO·DCM to yield the desired product **53** through a self-dimerization process. Concurrently, intermediate **14E** can react with **52** and DABCO·DCM to form intermediate **14F**, which then undergoes a second ring-expansion with DABCO·DCM to generate the desired product **53**.

**Scheme 20:**
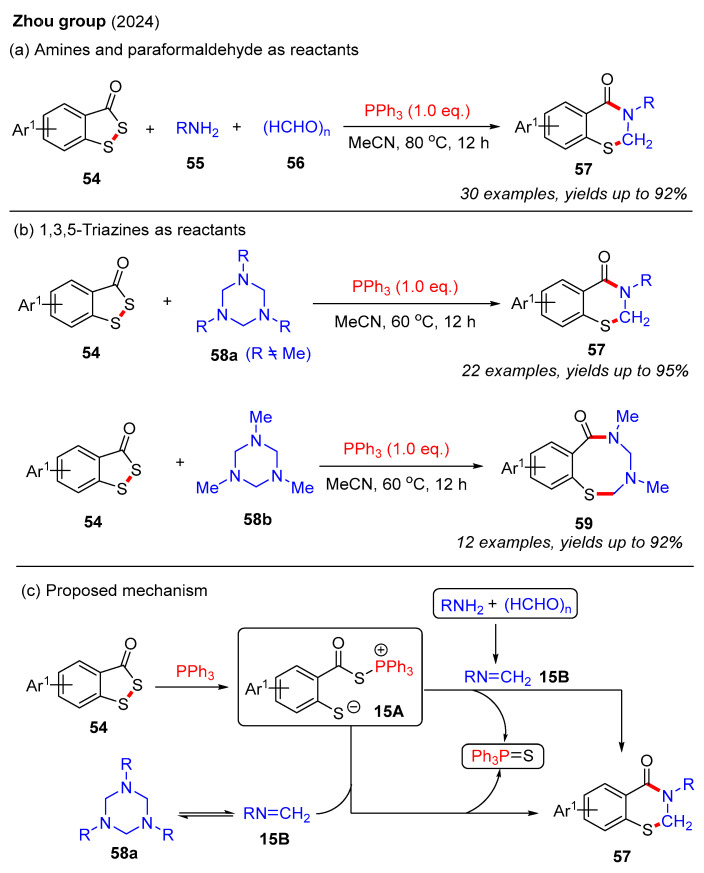
PPh_3_-mediated cyclization reaction of benzodithiol-3-ones for the construction of 2,3-dihydro-4*H*-benzo[*e*][1,3]thiazin-4-ones [58,59].

Recently, the Zhou group developed a PPh_3_-mediated three-component cyclization reaction of benzodithiol-3-ones, amines, and paraformaldehyde to construct 2,3-dihydro-4*H*-benzo[*e*][1,3]thiazin-4-ones (Scheme 20a) [58]. This method enabled the synthesis of various 2,3-dihydro-4*H*-benzo[*e*][1,3]thiazin-4-ones **57** in good yields using different amines. Furthermore, the same group also used the same strategy to achieve the synthesis of 2,3-dihydrobenzothiazin-4-ones employing benzodithiol-3-ones and 1,3,5-triazines as starting materials (Scheme 20b) [59]. To our surprise, when using 1,3,5-trimethyl-1,3,5-triazinane, only eight-membered benzothiadiazocin-6-ones **59** were isolated. In contrast, other substituted 1,3,5-triazinanes yielded the desired 2,3-dihydro-4*H*-benzo[*e*][1,3]thiazin-4-ones, likely due to steric effects. The plausible reaction mechanisms are outlined in Scheme 20c. Initially, the PPh_3_-mediated ring-opening of benzodithiol-3-ones **54** generates intermediate **15A**. Next, the imine intermediate **15B** is formed via the condensation of amine and paraformaldehyde or through the self-dissociation of 1,3,5-triazine. Finally, intermediate **15A** reacts with imine intermediate **15B** to form the desired product **57**, accompanied by the elimination of Ph_3_P=S.

## 4. Synthesis of 4H-Benzo[e][1,3]Thiazin-4-Ones via Intermolecular Pathways

**Scheme 21:**
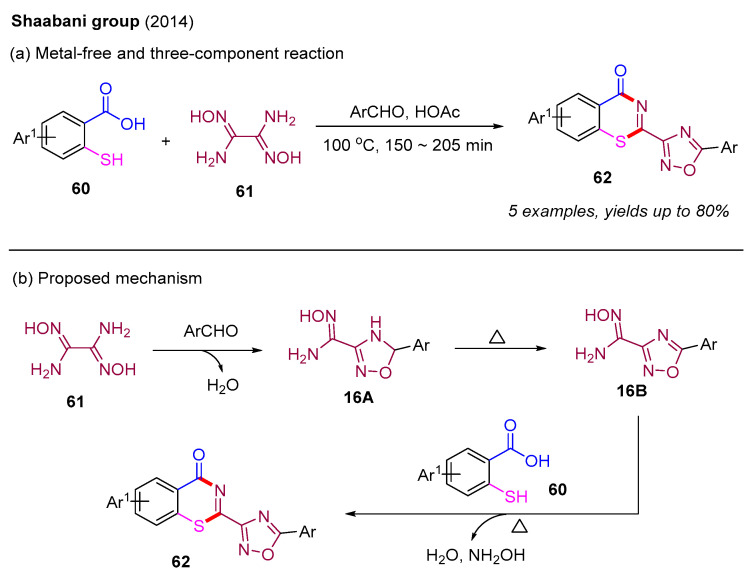
The synthesis of 4*H*-benzo[*e*][1,3]thiazin-4-one derivatives using 2-mercaptobenzoic acid, diaminoglyoxime, and aryl aldehydes [61].

Currently, there are limited intermolecular methods available for constructing 4*H*-benzo[*e*][1,3]thiazin-4-ones. Early synthesis methods involve the condensation of 2-mercaptobenzoic acids with various nitrile compounds [60]. In 2014, the Shaabani group prepared a series of 4*H*-benzo[*e*][1,3]thiazin-4-one derivatives using 2-mercaptobenzoic acid, diaminoglyoxime, and aryl aldehydes in HOAc solvent (Scheme 21a) [61]. In this process, different substituted aryl aldehydes (MeO and Cl) at the *para*-, *meta-*, or *ortho*-position were suitable, providing the desired products in good yields. Additionally, a plausible mechanism is also depicted in Scheme 21b. The initial condensation reaction between diaminoglyoxime **61** and aryl aldehyde in the solvent of HOAc provides the intermediate **16A**, which undergoes a subsequent oxidation to form the intermediate **16B**. Next, 2-mercaptobenzoic acid **60** reacts with the intermediate **16B** to produce the final product **62**.

**Scheme 22:**
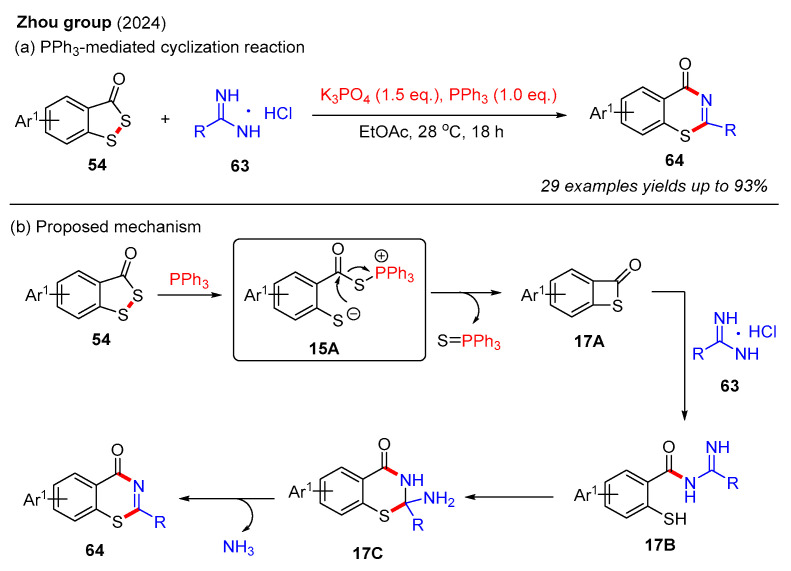
PPh_3_-mediated cyclization reaction of benzodithiol-3-ones and amidines for the construction of 4*H*-benzo[*e*][1,3]thiazin-4-ones [62].

Recently, the Zhou group reported a novel PPh_3_-mediated cyclization reaction between benzodithiol-3-ones and amidines, resulting in the efficient synthesis of 4*H*-benzo[*e*][1,3]thiazin-4-one (Scheme 22a) [62]. This method facilitated the preparation of a diverse range of 4*H*-benzo[*e*][1,3]thiazin-4-one in moderate to good yields through the use of various benzodithiol-3-ones and amidines. In this reaction, K_3_PO_4_ is a crucial base for activating amidines. It should be noted that the reduction of 4*H*-benzo[*e*][1,3]thiazin-4-one with NaBH_4_ and MeOH afforded 2,3-dihydro-4*H*-benzo[*e*][1,3]thiazin-4-ones in good yields. A plausible reaction mechanism for this process is described in Scheme 22b. Initially, benzodithiol-3-one **54** reacts with PPh_3_ to form intermediate **15A**, which is further converted into intermediate **17A** via intramolecular nucleophilic attack. Subsequently, the unstable intermediate **17A** is easily attacked by amidine **63** to generate intermediate **17B**, which then undergoes intramolecular cyclization to afford intermediate **17C**. Finally, the elimination of NH_3_ produces the desired product **64**.

## 5. Conclusions

In this review, we present a comprehensive overview of recent advancements in the synthesis of benzo[*d*]isothiazol-3(2*H*)-one and benzo[*e*][1,3]thiazin-4-one derivatives. The initial section outlines the intramolecular and intermolecular synthetic methodologies for benzo[*d*]isothiazol-3(2*H*)-ones. The intramolecular pathways primarily involve the formation of N–S bonds in 2-mercaptobenzamides or 2-alkylthiobenzamides through different methods, including thermal and electrochemical approaches. In contrast, intermolecular pathways are mainly achieved using 2-halobenzamides with S_8_, KSCN, and CS_2_ via transition-metal catalysis under thermal conditions. The second section primarily discusses the synthesis of 2,3-dihydro-4*H*-benzo[*e*][1,3]thiazin-4-ones via intermolecular strategies, which involve various sulfur-containing substrates (2-mercaptobenzoic acids, 2-mercaptobenzamides, 2-alkylthiobenzamides, benzoisothiazol-3-ones, and benzodithiol-3-ones) with carbon or amine sources. However, the intramolecular methods are only achieved by using 2-alkylthiobenzamides. The final section describes the synthesis of 4*H*-benzo[*e*][1,3]thiazin-4-ones, which can only be achieved via intermolecular pathways. Recent methods involve a three-component reaction among 2-mercaptobenzoic acid, diaminoglyoxime, and aryl aldehydes, as well as a PPh_3_-mediated cyclization reaction between benzodithiol-3-ones and amidines.

While some significant reports have been published, numerous aspects in this field still require further improvement. (1) The development of electrochemical or photochemical methods for the construction of heterocyclic compounds is currently a hot topic [63]. To date, only one electrochemical example for the synthesis of benzo[*d*]isothiazol-3(2*H*)-ones has been reported. Therefore, electrochemical and photochemical approaches may represent important future directions for the synthesis of these scaffolds. (2) It is worth noting that further studies on the synthesis of chiral 2,3-dihydro-4*H*-benzo[*e*][1,3]thiazin-4-ones are likely to become a key research focus. The aim of this review is to offer readers valuable insights and to inspire them to investigate innovative strategies for the synthesis of benzo[*d*]isothiazol-3(2*H*)-one and benzo[*e*][1,3]thiazin-4-one derivatives.

## Data Availability

Data are contained within the article.
